# Assessment of knowledge of COVID-19 among health care workers-a questionnaire-based cross-sectional study in a tertiary care hospital of India

**DOI:** 10.3934/publichealth.2021049

**Published:** 2021-08-26

**Authors:** Sukhbir Singh, Manjunath B Govindagoudar, Dhruva Chaudhry, Pawan Kumar Singh, Madan Gopal Vashist

**Affiliations:** 1 Associate Professor, Department of Hospital Administration, Pt. B. D. Sharma PGIMS, Rohtak, Haryana, India; 2 Assistant Professor, Department of Pulmonary & Critical Care Medicine, Pt. B. D. Sharma PGIMS, Rohtak, Haryana, India; 3 Sr. Professor and Head, Department of Pulmonary & Critical Care Medicine, Pt. B. D. Sharma PGIMS, Rohtak, Haryana, India; 4 Senior Professor and Unit Head, General Surgery, Pt. B. D. Sharma PGIMS, Rohtak, India

**Keywords:** Public Health Emergency of International Concern (PHEIC), pandemic, World Health Organization (WHO), Health Care Workers (HCW), COVID-19

## Abstract

**Background:**

Health Care Workers (HCW) are among the primary stakeholders and front liners in the fight against COVID-19. They are in direct contact with the patients as primary caregivers and, therefore, are at a higher risk of infection. This Pandemic offers a unique opportunity to explore the level of knowledge among ground-level HCWs during this global health crisis.

**Objective:**

We conducted this study to assess the knowledge and awareness among HCW regarding the COVID-19 Pandemic in a tertiary care hospital.

**Methods:**

It was a cross-sectional study done on HCW comprising faculty, senior residents, junior residents, demonstrators, and nursing staff of various specialties directly involved in the care of suspected/confirmed COVID-19 patients. A pretested questionnaire consisting of 20 questions was used as a study tool and was circulated through the digital platform.

**Results:**

There were a total of 437 respondents. In the subgroup analysis, the respondents in the age group of 55–64 years had a higher mean knowledge score, followed by the respondents in the age group of 18–24 years. For years of experience, the mean knowledge score varied from 13.89 (10–20 years of experience) to 13.83 (5–10 years of experience). The mean knowledge score was the highest for consultants (14.10), followed by Resident Doctors (13.96).

**Conclusions:**

This study has shed some critical clues for further research and interventions. Firstly, as health care workers are probably learning about COVID-19 from their practical exposure rather than formal teaching, it is pertinent to address this issue through well-planned formal sessions of training workshops and lectures.

## 1. Introduction

Coronavirus (CoV) infections cause illnesses varying in severity from the common cold to acute respiratory distress syndrome (ARDS) [1]. In the past, CoV infections have been causative agents in several epidemics/outbreaks, like SARS (severe acute respiratory syndrome) in 2002 and Middle East respiratory syndrome (MERS) in 2012 [2],[3]. The recent outbreak of a novel coronavirus (COVID-19) in Wuhan City, Hubei Province, China, has had considerable public health implications [4]. The rapid rise in the number of reported cases led the World Health Organization (WHO) to declare COVID-19 as a public health emergency of international concern. This was followed by the WHO declaring it as a *Pandemic* on Mar 11, 2020. Since its spread, an overwhelming number of new cases have been reported globally. Until this manuscript, over 5 million patients and more than 340 thousand deaths had been reported [5].

Similarly, since the reporting of the first case in India, there has been an exponential rise in the number of cases. The Ministry of Health and Family Welfare, Govt. of India, has reported over one lakh cases and more than 4000 deaths due to COVID-19 [6]. Despite the irregular behavior of the spread of COVID-19 [7], several prediction models have been used to plan for COVID-19 Pandemic using different approaches, like graph neural networks [8]. The Health Care Workers (HCW) are among the main stakeholders and front liners in the fight against COVID-19, and their involvement right from the grass root levels to the apex of critical care management guides the severity of the pandemic [9]. They are in direct contact with the patients as primary caregivers and, therefore, are at a higher risk of infection. The training material for awareness about COVID-19 for health care workers is available on the World Health Organization and Centers for Disease Control and Prevention (CDC) websites [10],[11]. In addition to this, most of the information is circulated through social media, the internet, and government circulars. Several workshops, online lectures, and webinars have been conducted during the initial stages of COVID-19 to spread knowledge and awareness among health care workers. In this regard, the COVID-19 Pandemic offers a unique opportunity to explore the level of expertise among ground-level HCWs during this global health crisis. COVID-19 is a new disease, and various aspects of the clinical and microbiological profile of the infection are coming up daily. Being a rapidly evolving situation, each medical institution needs to keep its health care workers updated about the virus.

On literature review, it was found that very few studies have been undertaken regarding the assessment of the knowledge of HCW about COVID-19. Thus, a study was planned to assess the current level of knowledge among doctors and nursing staff regarding novel coronavirus across all levels, i.e., from junior residents to faculty among doctors and staff nurses to nursing sisters among the nursing cadre in a tertiary care hospital. This study was also approved by the institute's scientific committee and ethics committee.

## 2. Methods

### 2.1. Aims & Objectives

To study the knowledge and awareness among HCW regarding the COVID-19 Pandemic in a tertiary care hospital.
To study the association between the level of knowledge and selected socio-demographic variables among the respondents.

### 2.2. Study setting

It was a cross-sectional study done on HCW comprising faculty, senior residents, junior residents, demonstrators, and nursing staff of various specialties directly involved in the care of suspected/confirmed COVID-19 patients at the Post Graduate Institute of Medical Sciences, Rohtak, Haryana, India (n = 505). The study was conducted in April and May 2020.

### 2.3. Study tool

The Questionnaire was prepared after extensive literature review and taking input from the material available on WHO and CDC websites. The Questionnaire was pretested by pilot testing among 20 subject experts. The information received during pretesting was reviewed, and the questionnaire was modified accordingly. Questions in this tool included those about socio-demographic variables of participants and awareness about various aspects of the novel Coronavirus. The main questionnaire consisted of a total of 20 questions about COVID-19. Of these, 18 questions assessed the knowledge regarding the novel Coronavirus (based on facts). In addition, there were 02 questions to get the opinion of the health care workers about the adequacy and the source of their information about COVID-19. The Questionnaire was circulated among the participants on a digital platform (SurveyMonkey^®^). The link was shared with each participant, and informed consent was taken from each participant on the digital platform. The link was active for a defined period, during which two reminders were sent if the questionnaire was not filled out, and after that, the link was disabled, and the survey was closed. The participants who failed to submit their replies were dropped from the study. The participants who did not consent to participate and those who responded to the questionnaire during pretesting were excluded from the study. The members of the Institute Scientific Committee and the Institute Ethics Committee were also excluded from the sampling frame.

### 2.4. Data collection and analysis

The individual response data was collected through a digital platform (i.e., SurveyMonkey^®^). The master data sheet was prepared on Microsoft Excel^®^, and the master data was checked to ensure that the same participants did not answer twice or more. It was found that each participant had responded only once, and after that, the data was transferred on statistical software to SPSS version 21. Out of a total of 20 questions, eighteen (18) were close-ended and were scored. Each correct response was given a score of one. The overall mean score (95% CI) was calculated for all the health care workers. Various statistical tests, i.e., Chi-square test, t-test, analysis of variance (ANOVA), were used, and category-wise subgroup analysis was done. The Knowledge differentials (i.e., mean knowledge score) of doctors and nursing staff were compared based on socio-demographic variables.

## 3. Results

All the above-mentioned health care workers on the roll of the selected departments were included in the sampling frame (n = 505). The category-wise detail of sample size is provided in Table 1.

**Table 1.** Detail of sample size (actual number and number of respondents) (n = 505).

S. No.	Designation	Total No. (n)	Total No. of Responses n (%)
1	Medical Faculty	49	29 (59.2)
2	Resident Doctors	230	221 (96.1)
3	Nursing Staff	226	187 (82.7)
Total		505	437 (87)

There was a total of 437 respondents. It was observed that 68% of participants were 25–34 years old, 63% were married, 69% were females, 48% were graduates, followed by postgraduates (44%), 55% were doctors, 90% were from Rohtak city, and 41% had experienced between 5–10 years, followed by less than five years of experience.

The mean knowledge score of the doctors was 13.98 (SD = 1.630, SE = 0.119), and the mean knowledge score of the nursing staff was 13.62 (SD = 1.690, SE = 0.107). The difference in mean knowledge score was not statistically significant (p = 0.87) (Table 2).

**Table 2.** Mean knowledge score of participants.

Total Score	Designation	N	Mean	Std. Deviation	Std. Error Mean	P valve = 0.87
	Nurses	187	13.62	1.630	0.119	
	Doctors	250	13.98	1.690	0.107	

The mean percentage score was 77% of the total achievable score. Knowledge scores varied from 28% to 100%, with a median of 78% (Standard Deviation = 9%).

In the subgroup analysis, the respondents in the age group of 55–64 years had a higher mean knowledge score, followed by the respondents in the age group of 18–24 years. With respect to years of experience, the mean knowledge score varied from 13.89 (10–20 years of experience) to 13.83 (5–10 years of experience). The mean knowledge score was the highest for consultants (14.10), followed by Resident Doctors (13.96). Females had a marginally higher score than males. It was found that the post-doctoral degree holder respondents had the highest mean score, followed by postgraduate respondents with a mean knowledge score of 13.85. The respondents working inward had the highest mean knowledge score (13.98), followed by respondents posted in the OPD area (13.73). The respondents residing outside Rohtak (urban) had the highest mean score (13.96), followed by respondents living in Rohtak city (13.84). The difference in the above-stated mean knowledge scores in various groups was not statistically significant (Table 3).

**Table 3.** Comparison of mean knowledge score of participants in various subgroups based on different socio-demographic variables.

Parameter	Mean	N	Std. Deviation	Median	Significance value (p-value)
Experience (years)					0.975
Less than 5 years	13.78	138	1.787	14.00	
5 to 10 years	13.83	180	1.716	14.00	
10 to 20 years	13.89	73	1.286	14.00	
More than 20 years	13.80	44	1.733	14.00	
Total	13.82	435	1.672	14.00	
Designation					0.077
Nurses	13.62	187	1.630	14.00	
Residents	13.96	221	1.665	14.00	
Consultants	14.10	29	1.896	14.00	
Total	13.83	437	1.672	14.00	
Residence					0.422
Rohtak City	13.84	390	1.704	14.00	
Rohtak Rural	12.88	8	1.458	13.00	
Outside Rohtak (Urban)	13.96	26	1.428	14.00	
Outside Rohtak (Rural)	13.73	11	1.191	14.00	
Age Groups					0.622
18 to 24 years	14.04	27	1.556	14.00	
25 to 34 years	13.80	297	1.757	14.00	
35 to 44 years	13.93	67	1.210	14.00	
45 to 54 years	13.50	30	1.796	13.50	
55 to 64 years	14.19	16	1.721	14.00	
Total	13.83	437	1.672	14.00	
Gender					0.214
Male	13.76	302	1.705	14.00	
Female	13.98	134	1.596	14.00	
Total	13.83	436	1.673	14.00	
Marital Status					0.050
Married	13.71	276	1.701	14.00	
Unmarried	14.03	161	1.606	14.00	
Total	13.83	437	1.672	14.00	
Occupation					0.069
Doctor	14.01	245	1.679	14.00	
Nurse	13.61	187	1.645	14.00	
Others	13.60	5	0.894	14.00	
Total	13.83	437	1.672	14.00	
Place of Posting					0.274
Ward	13.98	241	1.531	14.00	
ICU	13.66	87	1.904	14.00	
OPD	13.73	30	1.741	14.00	
Others	13.65	77	1.738	14.00	
Total	13.84	435	1.665	14.00	
Educational Qualification					0.942
Graduate	13.81	209	1.655	14.00	
Postgraduate	13.85	191	1.730	14.00	
Post-Doctoral or equivalent	14.10	10	1.524	14.00	
Others like Diploma	13.73	26	1.485	14.00	
Total	13.83	436	1.672	14.00	

For open-ended introductory questions, when participants were asked whether they feel that they have sufficient knowledge about COVID-19, 335 (77%) responded that they had enough knowledge about the novel Coronavirus. In contrast, only 11.4% answered that they did not have adequate knowledge. However, more nurses than doctors believed that they had sufficient knowledge about COVID-19 (p = 0.016). In subgroup analysis according to marital status, a higher number of unmarried people thought that they had insufficient understanding of COVID-19 compared to married (p < 0.001). Regarding their source of knowledge for COVID-19, 220 (50.34%) participants responded that social media and the internet were their primary source of information, whereas 192 (43.94%) responded that government circulars were the primary sources of information. Interestingly, social media was the primary source of information of COVID-19 for Doctors, whereas government circulars were the main source of information for nurses (p = 0.019).

On individual question wise analysis, it was found that knowledge of the participants for questions like country of origin of the novel corona viral infection, its incubation period, availability of its vaccine in market, knowledge about its first line of treatment, people who are at higher risk of getting novel corona viral infection and persons who should be included in contact tracing and sampling sites for detection of Coronavirus was very good (score more than 90%). The knowledge was good about other questions, like the isolation of the exposed persons, myths about prevention of novel coronavirus infection, its fatality, its least common presentations, duration of quarantine of exposed persons, intermediate host, and the official name of the recent strain of novel coronavirus (score between 60 and 90%). However, a substantially higher proportion of males knew the right strain compared to females (p = 0.001). The significantly higher number of nurses knew the latest term for COVID-19 as compared to doctors (p < 0.001), However, knowledge was average for some questions, like the precautionary measure for preventing infection from novel coronavirus, its mode of transmission, and the declaration of PHEIC by WHO (score less than 50%). A significantly higher number of males thought that this was the first time WHO had declared a PHEIC compared to females (p = 0.01), and a considerably higher number of nurses wrongly believed that was the first time WHO had declared a Public Health Emergency of International Concern (PHEIC) (p < 0.001) as compared to doctors. In an astounding revelation, a significantly higher number of nurses believed that staying at home till the COVID-19 epidemic was over was a correct precautionary measure (p < 0.001).

Similarly, the significantly higher proportion of males believed staying at home until the COVID-19 outbreak is a recommended precautionary measure (p = 0.003). In addition, a substantially higher proportion of respondents posted inwards considered that staying at home till the COVID-19 outbreak is over is the correct method of prevention (as compared to those posted at other places) (p < 0.001). But the higher number of nurses compared to doctors believed that the recommended quarantine period was 21 days (p < 0.001) instead of 14 days. The significantly lower number of respondents posted inward knew that the duration of quarantine was 14 days and not 21 days (p < 0.001).

## 4. Discussion

All healthcare workers must be aware of various aspects of the novel coronavirus, later named COVID-19 by the WHO. As the disease is recent, the knowledge status about it is dynamic and rapidly evolving. Knowledge and awareness of COVID-19 among HCW has not been evaluated extensively. Therefore, this study was conducted among HCW of a tertiary care hospital in a developing country to map their knowledge of COVID-19.

We found that our study's overall mean knowledge score was 77% of the total achievable score on knowledge scoring. It was less than the scores quoted in studies conducted in China [12],[13], where around 89% of the health care workers had sufficient knowledge about COVID-19. This difference in knowledge score may be attributed to the fact that the novel Coronavirus was reported in China for the first time. This study has generated some crucial differentials in the knowledge scores. The respondents above 50 years of age, consultants, and participants with higher experience and educational qualifications scored marginally better. Better knowledge scores in some categories compared to others could be because of greater exposure to the subject in the former compared to the latter. This exposure can be due to better teaching and training or/and practical exposures.

There were several revelations in our analysis that were striking, like, in our study, a higher number of nurses than doctors believed that they had sufficient knowledge about COVID-19. This finding agrees with a study conducted in China [12] where doctors showed higher knowledge scores than nurses.

A significantly higher number of nurses and respondents with only a graduation degree believed COVID-19 could be fatal in as many as 80% of the cases (p < 0.001). In our study, 220 (50%) participants stated that social media and the internet were their sources of information about the novel coronavirus, more so for doctors than nurses. Similarly, it was found that social media was the primary source of information among unmarried respondents than married ones, for whom government circulars were the primary source of information. These findings were similar to those of other studies, like in a study carried out in China [14], where 98% of the respondents believed that official government websites and social media were their primary source of information about COVID-19. This finding of the source of COVID-19 information is alarming, as widely unauthenticated information is available on social media and the internet, which may create confusion and anxiety among HCW. This argument is supported by various other studies [4],[15]–[17].

In the current study, the knowledge about the transmission of this disease was average (49%) among the respondents. This finding was in agreement with the results of a study conducted in China [14], where it was found the knowledge of respondents about the transmission of the disease was poor. On the contrary, the knowledge about the incubation period in the current study was very good, and the same was poor in the China study [14]. This difference may be attributed to the widely circulated government instructions of 14-day quarantine for Indian citizens brought back from other countries. There are theories related to the role of environmental pollution in COVID-19 transmission/diffusion is coming up. The data in support of this hypothesis comes mainly from Italy following its first wave of experiences [18]–[20].

In this study, 18% of respondents were aware of the incorrect precautionary measure for preventing infection from novel coronavirus. This finding is in agreement with a study conducted in China [12]. Additionally, it was found that overworked HCW washed their hands less frequently than their colleagues who were not overworked.

In an astounding revelation, many participants believed that staying at home during the COVID-19 epidemic is a correct precautionary measure. This was a surprising finding as the alternatives were frequent hand washing and avoidance of close contact with a confirmed case. Despite multiple and frequent notifications and awareness messages, a low score on this question states that more intense and individual training sessions should be conducted predominantly for nurses and other subgroups where scores have been lacking.

In the current study, it was observed that 81% of the health care workers were aware of the quarantine period of exposed subjects/people. When these findings were compared to previous studies, the overall score was higher than the score recorded in other studies (66% in a study [21] conducted in Ho Chi Minh City).

This study found that the least probable presentation of COVID-19 was known to doctors more than to nurses, which was expected. In addition, the higher number of HCW posted in ICU knew the least common presentation of COVID-19. This was also anticipated as most rare manifestations were seen in patients admitted to intensive care units. In contrast, the significantly higher number of respondents with only a graduation degree believed severe ARDS is the least probable presentation of COVID-19.

A significantly higher number of nurses and respondents with only a graduation degree believed COVID-19 could be fatal in as many as 80% of the cases. This could have been an outcome of COVID-19 anxiety and uncensored information sharing. Such kinds of fear cause mental trauma among HCW, which might impact their functionality and performance. The revelation of this finding should incite an urgent structured awareness program among nurses who are posted in COVID-19 management areas. Contrasting results were published in the studies from China. Like in study [21] conducted in Ho Chi Minh City, 73% of the respondents reported that only patients with underlying chronic diseases are at a higher risk of infection and death (79.2%).

This study has given insight into the various domains of COVID-19, where respondents have performed well and those where significant gaps still exist. It is encouraging to note that in our study, respondents scored more than 90% regarding various aspects of COVID-19. But at the same time, several jarring findings were also noticed, like the lack of awareness about mortality rates and preventive measures. Especially in some focused subgroups, a poor result on these topics is alarming and needs to be addressed on a priority basis.

### 4.1. Strength of this study

The major strength of our study is that our sample size was representative of all health care workers, i.e., medical doctors (i.e., consultants, Senior Resident and Junior Resident doctors, demonstrators) and nursing staff (i.e., Nursing Sisters and Staff Nurses) across all departments (i.e., General Medicine, Pediatrics, Emergency, ICU, Respiratory Medicine, Pulmonary and Critical Care Medicine, ENT, Microbiology, etc.) who were directly involved in the treatment of COVID-19 and among them we were able to get responses from more than 80% HCW. Thus, it will serve as an essential yardstick for knowledge of novel coronavirus (COVID-19) among health care workers in a hospital setting, particularly in developing countries.

### 4.2. Limitations of this study

There were some significant limitations to our study, like it was a single center-based study, and other multiple centers were not evaluated. Most of the respondents had undergone similar training sessions, so the uniformity could have been attributed to the same. Also, a large majority of respondents are 25–34 years old, and hence selection bias cannot be excluded. Over the course of several waves, the impact of COVID-19 and isolation have been studied in various studies. It would have been interesting to collect data on such psychological aspects of the disease, like Sonza et al. in their multicentric study [22].

## 5. Conclusions

The subcategory analysis scores and differences generated in the study should be absorbed with care, as the influence of information sources was not evaluated in our research. Nonetheless, this explorative analysis has shed some important clues for further research and interventions. The study found that the knowledge score regarding different domains of the disease had large variations. Hence, it should be made a compulsory topic of induction training for health care workers before posting to the COVID-19 treatment area.
